# The neural correlates of agrammatism: Evidence from aphasic and healthy speakers performing an overt picture description task

**DOI:** 10.3389/fpsyg.2014.00246

**Published:** 2014-03-21

**Authors:** Eva Schönberger, Stefan Heim, Elisabeth Meffert, Peter Pieperhoff, Patricia da Costa Avelar, Walter Huber, Ferdinand Binkofski, Marion Grande

**Affiliations:** ^1^Section Neurological Cognition Research, Department of Neurology, Uniklinik RWTH AachenAachen, Germany; ^2^Department of Psychiatry, Psychotherapy and Psychosomatics, Uniklinik RWTH AachenAachen, Germany; ^3^Research Centre Juelich, Institute of Neuroscience and Medicine (INM-1)Juelich, Germany

**Keywords:** fMRI, spontaneous language production, syntax, morphology, agrammatism, functional reorganization, aphasia

## Abstract

Functional brain imaging studies have improved our knowledge of the neural localization of language functions and the functional reorganization after a lesion. However, the neural correlates of agrammatic symptoms in aphasia remain largely unknown. The present fMRI study examined the neural correlates of morpho-syntactic encoding and agrammatic errors in continuous language production by combining three approaches. First, the neural mechanisms underlying natural morpho-syntactic processing in a picture description task were analyzed in 15 healthy speakers. Second, agrammatic-like speech behavior was induced in the same group of healthy speakers to study the underlying functional processes by limiting the utterance length. In a third approach, five agrammatic participants performed the picture description task to gain insights in the neural correlates of agrammatism and the functional reorganization of language processing after stroke. In all approaches, utterances were analyzed for syntactic completeness, complexity, and morphology. Event-related data analysis was conducted by defining every clause-like unit (CLU) as an event with its onset-time and duration. Agrammatic and correct CLUs were contrasted. Due to the small sample size as well as heterogeneous lesion sizes and sites with lesion foci in the insula lobe, inferior frontal, superior temporal and inferior parietal areas the activation patterns in the agrammatic speakers were analyzed on a single subject level. In the group of healthy speakers, posterior temporal and inferior parietal areas were associated with greater morpho-syntactic demands in complete and complex CLUs. The intentional manipulation of morpho-syntactic structures and the omission of function words were associated with additional inferior frontal activation. Overall, the results revealed that the investigation of the neural correlates of agrammatic language production can be reasonably conducted with an overt language production paradigm.

## Introduction

In natural continuous speech production, multiple single-words are combined into larger units to convey a message in a highly automated process. For each utterance the relevant concepts and their associated lemmas in the mental lexicon are retrieved. Besides the word, its morpho-syntactic features such as number, tense, grammatical gender and word class as well as the syntactic frame defining its possible structural environment are activated. In a morpho-syntactic encoding process the lexical items with their specific constraints are combined into a syntactic structure and an utterance is generated (Vosse and Kempen, 2000; Levelt, 2001; Hagoort, 2003, 2005; Indefrey and Levelt, 2004; Menenti et al., 2012). The term *morpho-syntax* thus depicts different aspects of a sentence structure as its complexity (compound or simple sentences), the completeness, and the correct use of function words and flexional elements (morphology).

### Agrammatism in aphasia

Aphasic speakers with agrammatism show deficits in the morpho-syntactic encoding in language production and perception (e.g., Kolk and Heeschen, 1990; Friedmann and Grodzinsky, 1997; Kolk, 1998; Rochon et al., 2000; De Roo et al., 2003; Prins and Bastiaanse, 2004; Lee et al., 2008; Bastiaanse et al., 2009). Their language production is characterized by the omission and substitution of function words and flexional elements. Simple sentence constructions without subordination are used that are often incomplete (e.g., due to a missing verb). Individual differences in symptoms are due to the size and location of the lesion and to the duration and severity of the aphasia. Neurological accounts often related agrammatic symptoms to lesions in Broca's area but also to lesions in other perisylvian regions in the language dominant hemisphere (Cappa, 2012). Due to those findings as well as an increasing number of neuroimaging studies (see below), Broca's region is awarded a crucial role in morpho-syntactic processing although the inferior frontal gyrus (IFG) has been associated with different linguistic and non-linguistic functions since then (see e.g., Hagoort, 2005; Caplan, 2006; Santi and Grodzinsky, 2007).

The neural mechanisms that result in agrammatic language production after a lesion in language relevant areas remain widely unknown. There are different ways to approach those: First, structure-function mapping of morpho-syntactic processing in healthy individuals constitutes the basis to interpret results from agrammatic brain damaged speakers. Second, agrammatic-like speech behavior can be induced in healthy speakers to study the underlying linguistic or functional processes. Third, brain imaging experiments with agrammatic speakers reveal insights into the functional reorganization of language and the neural mechanisms underlying agrammatism.

### Functional neuroimaging of morpho-syntactic processing in healthy speakers

With respect to structure-function mapping (approach 1) many neuroimaging studies have shown correlates of single aspects of morpho-syntactic processing in the neurologically normal brain. Morpho-syntactic processes on word-level (e.g., determining word categories, gender processing, and inflection of verbs) have often been localized in the left pars opercularis and pars triangularis of the IFG in overt production and covert tasks (e.g., Heim et al., 2002, 2003; Indefrey et al., 2004; Longoni et al., 2005; Heim, 2008). Other areas like the superior and middle frontal gyri and posterior parietal regions are likely to play a role in the grammatical processing in language comprehension and production too (e.g., Miceli et al., 2002; Kielar et al., 2011). Results on the processing of morpho-syntactic violations stem from language comprehension tasks only that described activation in the left pars opercularis (Heim et al., 2010), the left posterior frontal operculum, the left anterior superior temporal gyrus (STG) (Friederici et al., 2003), and the middle frontal gyrus (MFG) (Indefrey et al., 2001b).

Fewer results exist from experiments on morpho-syntactic processing in multi-word speech production like sentence or even text level. Sentence production is supported not only by the left inferior frontal but also the middle and superior temporal gyrus, the inferior and superior parietal lobule (e.g., Haller et al., 2005; Vigneau et al., 2006, 2011; Menenti et al., 2011, 2012). Activation in the pars opercularis of the left IFG as well as in the left middle and superior frontal cortex was reported for phrase structures with higher syntactic demands compared with simpler phrase structures in language production (Den Ouden et al., 2008) and comprehension (Ben-Shachar et al., 2004; Friederici et al., 2006). Compound sentences with embedding have an increased complexity compared to simple sentences. In a comprehension task, the processing of complex sentences with embedding compared to those without was associated with activation peaks in the left IFG, the bilateral temporo-parietal cortices, bilateral MTG and precuneus (Shetreet et al., 2009). In an overt picture description task the number of produced complex sentences with embedding was positively correlated with activation in the left posterior middle temporal gyrus (pMTG) and the right posterior temporal sulcus (Kircher et al., 2005).

In sum, the results from brain imaging studies investigating effects of complexity, completeness, and morphological processing in sentence production or even spontaneous speech are rare and often stem from comprehension or covert processing tasks. But the interpretation of findings on the functional neuroanatomy of agrammatism needs results on the neural basis of unimpaired morpho-syntactic language production in healthy speakers as a point of origin, which shows brain areas that might have been associated with grammatical functions in language production before brain injury.

### Functional neuroimaging of agrammatic-like speech production

Recently, as a second approach, some studies induced aphasia-like speech behavior in healthy controls to study the underlying linguistic or functional processes (Meffert et al., 2011; Grande et al., 2012). De Roo et al. (2003) asked participants to describe pictures with a limited number of words (2 or 3) per utterance and compared these to the descriptions of agrammatic speakers. They showed a high agreement of used morpho-syntactic structures in both groups (e.g., overuse of finiteness omission). These results were replicated with German speakers (Hoffschildt, 2003). The authors concluded that healthy speakers reduce their utterance length in a comparable morpho-syntactic manner as agrammatic speakers in an appropriate task. Indefrey et al. (2001a, 2004) used a very similar paradigm in a functional imaging study. Again healthy participants had to describe scenes with varying demands in morpho-syntactic structures by limiting the utterance length. They found activation in the left caudal Broca's area and the adjacent Rolandic operculum for morpho-syntactic encoding in speech production. The strength of activation was positively correlated with the morpho-syntactic demands of the utterances. Grande et al. (2012) reported a lack of significant involvement of left area 44 when syntactically incomplete utterances (due to a missing verb or object) were compared to complete utterances elicited in a picture description task in healthy speakers.

By combining the methods of Indefrey et al. (2001a), De Roo et al. (2003), Meffert et al. (2011) and Grande et al. (2012) in a new fMRI paradigm, it should be possible to study the underlying functional neuroanatomy of agrammatic-like symptoms induced during a picture description task in a group of healthy speakers. The results might not only reveal areas specifically involved in the concerned morpho-syntactic processes but furthermore they could show, whether agrammatism in aphasia and provoked agrammatic behavior in healthy speakers share a common neural basis (see Heim, 2013 for a discussion on simulation of pathological symptoms in healthy speakers).

### Functional reorganization and neuroimaging of agrammatism in aphasia

As a third approach, brain imaging experiments with agrammatic participants might reveal insight into the functional reorganization of language and the neural mechanisms underlying agrammatism. Functional imaging studies with aphasic participants described a reorganization in the lesioned speech-dominant as well as in in the contralesional hemisphere. In the affected hemisphere, activation showed in preserved areas within the task-specific language network as well as in structures not recruited in normal language processing during various tasks like lexical decision and word repetition (Heiss et al., 1999; Abo et al., 2004; Specht et al., 2009; Turkeltaub et al., 2011). Other authors stressed the importance of activation in perilesional tissue that goes along with enhanced language performance in the course of recovery by possibly taking over altered functions of the lesioned region (Cao et al., 1999; Warburton et al., 1999; Rosen et al., 2000; Perani et al., 2003; Fernandez et al., 2004; Saur et al., 2006; Postman-Caucheteux et al., 2010). Additional activation in the unaffected hemisphere has often been reported (Saur et al., 2006; Turkeltaub et al., 2011), especially in homolog areas. Whether the right hemisphere can reorganize in order to assume functions of the lesioned hemisphere and thus facilitate recovery even if they possibly are computationally less efficient in language processing (Heiss et al., 1999, 2003; Thulborn et al., 1999; Abo et al., 2004; Naeser et al., 2004; Winhuisen et al., 2005) or whether this compensational activation constrains recovery due to interhemispheric inhibition (Cao et al., 1999; Postman-Caucheteux et al., 2010; Szaflarski et al., 2013) has been subject to debate. This latter view is supported by studies with inhibitory right hemisphere transcranial magnetic stimulation (TMS) of the intact right pars triangularis that resulted in improved naming in several case studies (Martin et al., 2009; Hamilton et al., 2010; Barwood et al., 2011; Naeser et al., 2011; Turkeltaub et al., 2012) and in improved naming, picture description and spontaneous speech performance in a single case study (Hamilton et al., 2010, but see Martin et al., 2009 for a different effect). At the same time inhibitory TMS at the right pars opercularis has been reported to disrupt naming in aphasic speakers (Hamilton et al., 2010; Naeser et al., 2011). As not all participants as well as the different brain areas responded the same to TMS, the functional role of the right hemisphere might differ individually and the assumption of interhemispheric inhibition might not apply for all regions (Winhuisen et al., 2007; Hamilton et al., 2010; Turkeltaub et al., 2012). Besides linguistic processes, right hemisphere activation in aphasic speakers has been attributed to compensational and non-linguistic processes like effort or executive control that are recruited when processing load is high (Blank et al., 2003; Price and Crinion, 2005; Postman-Caucheteux et al., 2010; Van Oers et al., 2010). Compensatory strategies used by an aphasic speaker to overcome the processing difficulty might unsuccessfully result in aphasic errors.

The specific impact of activation in right hemisphere regions on agrammatic speech production in aphasia has not been subject to study. Starting from the results discussed above, some right hemisphere areas might support recovery in aphasia and agrammatism, although perhaps with a restricted compensatory potential leading to morpho-syntactic errors or syntactically reduced sentence structures (proto-language, Springer et al., 2000; Code, 2011), some might interfere and others might play no causal role (Winhuisen et al., 2007; Turkeltaub et al., 2011, 2012). This could be contingent on the reason for the increased right hemispheric activation: whether they are related to transcallosal disinhibition or to compensatory strategies (Price and Crinion, 2005) with the latter supporting recovery. Besides, right hemisphere recruitment seems to depend on the size and site of the lesion, time post onset and the severity of the aphasia (Blank et al., 2003; Heiss et al., 2003; Abo et al., 2004; Fernandez et al., 2004; Saur et al., 2006; Crosson et al., 2007).

Activation that goes along with aphasic errors might give further insights into the role of both hemispheres in successful reorganization especially when contrasted with correct language production. However, most studies on functional recovery in aphasia either used language tasks that the aphasic participants could perform correctly or did not distinguish between correct and inaccurate responses. Previous fMRI studies on aphasic errors are limited to incorrect naming responses and unsolved lexical retrieval that were associated with left and/or right-sided-contralesional activation patterns (Fridriksson et al., 2009; Postman-Caucheteux et al., 2010; Tillmanns et al., 2011). Naeser et al. (2004) compared the activation of four non-fluent aphasic speakers' speech production with that of a healthy control group in a picture description task. In the aphasic speakers, a relatively higher activation in right hemisphere areas was associated with their agrammatic and hesitant speech. This was interpreted as a dysfunctional process causing the hesitant speech. The neural mechanisms associated with characteristic impaired morpho-syntactic aspects in agrammatism like completeness, complexity and morphology have not been investigated so far.

In sum, it remains unclear if agrammatic language production is accompanied by enhanced or reduced left and/or right hemispheric activation, within the normal language network or in a distinct area. These questions could be investigated by studying the specific activation patterns associated with agrammatic errors when compared with unimpaired language production in aphasic speakers. This knowledge would contribute to the complex picture of functional reorganization processes and to the neural correlates of agrammatism and eventually might guide subsequent brain stimulation studies.

### Aim and hypotheses

The exploration of morpho-syntactic capacities and underlying processes requires more than the processing of single words (Blank et al., 2002). Although they can be observed best on sentence level or even in spontaneous speech, only few studies have detached from explicit meta-linguistic tasks on word-level and studied agrammatic speech in overt language tasks which are particularly promising for patient studies (Foki et al., 2008). Investigating continuous language production in an fMRI-paradigm is possible (e.g., Naeser et al., 2004; Kircher et al., 2005; Troiani et al., 2008). Grande et al. (2012) and Tillmanns et al. (2011) used an open picture description task to relate brain activation to linguistic phenomena like word-finding difficulties in a group of healthy speakers and in a single case aphasic participant. Those studies explicitly looked at activation in the brain when the speech errors occurred and contrasted them with undisturbed language.

Because the neural correlates of morpho-syntactic encoding and agrammatic errors in continuous language production have not been studied yet, the aim of this fMRI study was to investigate the activation patterns underlying the aspects completeness, complexity and morphology in normal and disordered grammatical processing in an overt picture description task. Based on the results of De Roo et al. (2003) and Hoffschildt (2003) the morpho-syntactic demands of produced utterances were varied in a group of healthy speakers using a newly designed paradigm. Agrammatic-like as well as unimpaired language production were captured and contrasted (experiment 1: combination of the approaches 1 and 2). The results were compared to structure-function mapping of morpho-syntactic processing in five agrammatic single cases (experiment 2: approach 3). Again, agrammatic language production was contrasted with unimpaired language production. Although there has been no comparable study so far, the literature described above permits some expectations concerning possible activation patterns. Our hypotheses were:

- The 3-word paradigm is suited for inducing agrammatic language production in healthy speakers.
- The reduction of morpho-syntactic structures [complete vs. incomplete/complex vs. simple clause-like units (CLU)] is associated with reduced effects in the well-known predominantly left hemisphere network reported for syntax processing including pars triangularis and pars opercularis of the IFG, the Rolandic operculum and posterior temporal regions. This is applicable for the production as well as the planning of utterances.
- Morphological errors in the healthy speakers are correlated with activation in the left pars triangularis and pars opercularis of the IFG and in the left MFG as well as in in additional brain areas subserving monitoring processes.
- In the agrammatic speakers, effects for completeness, complexity and morphology show in similar left hemisphere activation networks as the respective effects in the healthy speakers with activation in preserved and perilesional tissue.
- Agrammatic language production goes along with stronger effects in the areas described above when compared with unimpaired language production and language production in the healthy speakers.
- Agrammatic language production is associated with enhanced right hemisphere activations when compared with unimpaired language production and language production in the healthy speakers.

## Materials and methods

### Participants

Participants gave their written informed consent before participating in the study, that was approved by the ethics committee of the Medical Faculty RWTH Aachen University (reference no. EK 040/07).

#### Experiment 1: healthy speakers

Twenty subjects with no history of neurological, psychiatric or speech and language disorders participated in the study. All were native German speakers and presented with normal or corrected-to-normal vision. Five subjects had to be excluded from the study due to technical problems during fMRI image acquisition. The final experimental group consisted of 6 female and 9 male participants in the age range of 23 to 67 years (mean 43.53). Handedness was assessed by the Edinburgh Handedness Inventory (Oldfield, 1971). All participants were right-handed with a laterality quotient of at least 60% (mean 89.27).

#### Experiment 2: agrammatic aphasic speakers

**Figure 1:**
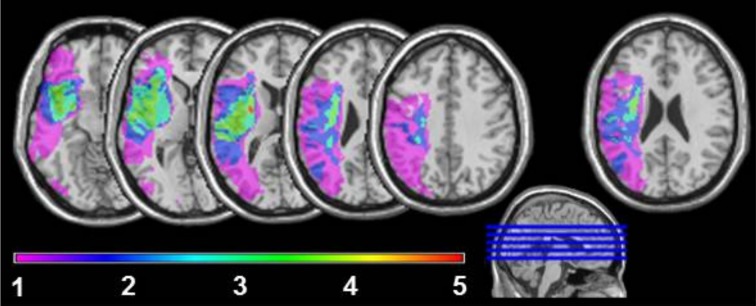
**Lesion sites overlapping in 1, 2, 3, 4, or 5 of the agrammatic speakers**. For the very right axial slice, first a lesion overlay of 11 non-agrammatic aphasic speakers was plotted. Next the brain areas, where at least 6 lesions overlapped, were subtracted from the overlay plot of lesion sites in the five agrammatic speakers to adjust for areas with higher anatomical vulnerability.

Five aphasic participants (1 woman), showing agrammatic speech production and being able to perform the picture description task using utterances above single-word level, participated in the study. To avoid movement artifacts, participants with severe apraxia of speech were not included. Further exclusion criteria were a history of psychiatric disease, premorbid language disorders or any contraindication for fMRI. The age ranged from 21 to 48 years (mean 39.2). All aphasic speakers presented with chronic moderate to mild Broca's aphasia [30–51 months post onset (mean 39.4)] secondary to a left hemisphere cerebral vascular accident affecting different brain regions with lesion foci in the insula lobe, inferior frontal, superior temporal and inferior parietal areas. The lesion overlap in Figure 1 reveals that all five lesions overlap only in part of the insula lobe and the putamen. The non-fluency of the aphasia was classified by means of the Aachen Aphasia Test (AAT, Huber et al., 1983) spontaneous language syntax scale score 2 in all five aphasic participants. Descriptive and clinical information is given in Table 1.

**Table 1:** **Aphasic speakers' (A1–A5) demographic and clinical data**.

	**A1**	**A2**	**A3**	**A4**	**A5**
Sex	Female	Male	Male	Male	Male
Age (years)	37	43	47	21	48
Months post stoke	41	51	30	31	44
LQ	100	80	48	100	100
Handedness	Right	Right	Ambidextrous	Right	Right
Etiology	Ischaemic stroke	Ischaemic stroke	Ischaemic stroke	Ischaemic stroke	Ischaemic stroke
Aphasia severity	Mild	Moderate-mild	Mild	Moderate	Mild
Aphasia syndrome	Broca	Broca	Broca	Broca	Broca

*LQ, laterality quotient*.

### Stimuli

**Figure 2:**
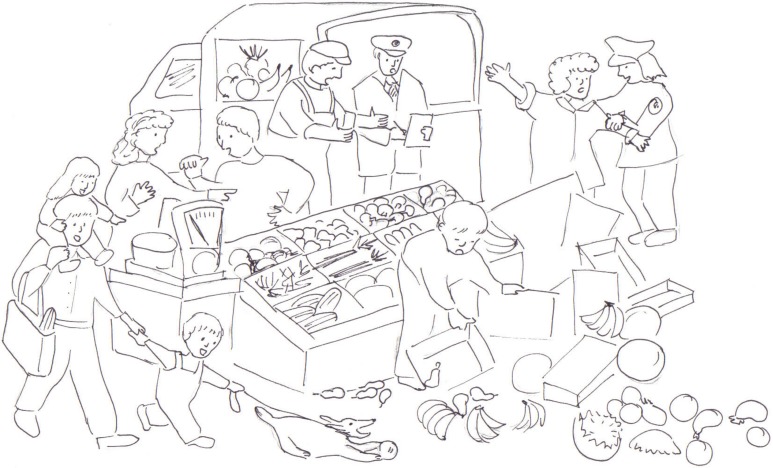
**Example of stimulus picture**.

In experiments 1 and 2, spontaneous language was elicited using 9 black and white line drawings showing complex scenes like a market (Figure 2) with various people, objects and actions (see Meffert et al., 2011 for an overview over all pictures). They were controlled for the number of main propositions. A discriminant analysis as well as a repeated measures ANOVA revealed no significant differences in linguistic parameters [absolute number of items, words, open class words and CLUs, proportion of open class words on all words and mean length of utterances (MLU)] between each of the nine pictures and, respectively, the other eight pictures (Meffert et al., 2011). Earlier studies have shown the pictures to be suitable for eliciting descriptions of about 3 min in healthy as well as aphasic participants (Meffert et al., 2011; Tillmanns et al., 2011; Grande et al., 2012).

### Experimental design

During fMRI the stimuli were presented via goggles (VisuaStimTM, Resonance Technology Inc., CA, USA) using Presentation 11.0 software (Neurobehavioral Systems, Albany, CA, USA). Each picture appeared in randomized order for 3 min, in which the participants described in detail what was happening in the picture. All pictures were followed by a blank screen for 55 s and subsequently a fixation cross for 5 s, to focus the participants' attention on the upcoming picture. During scanning verbal output was recorded using the headphone's microphone and Audacity 2.0.1, an open source program for recording and editing sound.

#### Experiment 1: two conditions

There is a relation between utterance length and syntactic structure (De Roo et al., 2003). Therefore, in five of the pictures the healthy participants were instructed to use only utterances consisting of three words to reduce the morpho-syntactic elaborateness of the speech output. The written instruction *Max. 3 Wörter* (three words at most) was shown 5 s before the 1st, 3rd, 5th, 7th, and 9th picture appeared (3W-condition). Five seconds before the 2nd, 4th, 6th, and 8th picture the instruction on the screen was *Natürlich beschreiben* (decribe naturally, NAT-condition). Before scanning both conditions were practiced with one additional picture with the healthy participants. Feedback was given on the way of description to avoid deictic descriptions and labeling. Furthermore, it was made sure that the participant was able to accomplish the descriptions in the 3W-condition properly.

#### Experiment 2: one condition

The aphasic participants were asked to describe the nine pictures without an additional instruction concerning the utterance length. They were familiarized with the stimuli and the task demands in four sessions outside the scanner where each picture was described twice in total by the aphasic participants.

### fMRI data acquisition

Imaging was performed on a 3 Tesla Scanner (Philips Achieva 3.0 T X-series) using a Philips SENSE head coil eight Elements. For each participant 535 functional scans were acquired in 42 sagittal slices of 3.75 mm thickness without interslice gap covering the whole brain using a fast-field-echo gradient EPI sequence, a repetition time (TR) of 4000 ms, echotime of 30 ms, a flip angle (FA) of 90° and a 240 mm field of view (FOV). The in-plane resolution was 3.75 × 3.75 mm. The structural T1-weighted MP-RAGE images were taken with a 3D FFE sequence (180 slices, TR 1000 ms, TE 4.6 ms, FA 8°, FOV 256 mm, resolution 1 × 1 × 1 mm).

### Behavioral data analysis

**Figure 3:**
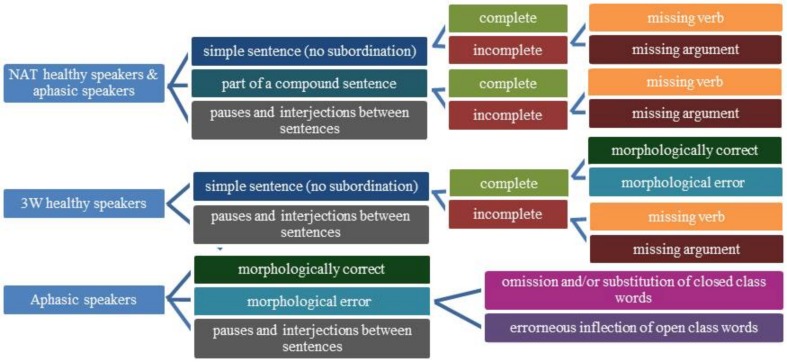
**Categories for classification of clause-like units**.

Speech recordings were transcribed using ASPA (Aachener Sprachanalyse, Huber et al., 2005). Within ASPA, transcription includes the classification of CLU, defined as a syntactically and/or prosodically marked part of speech referring to a proposition and containing one verb at most (Grande et al., 2008). Subordinated and embedded CLUs in compound sentences were identified. Because the intended morpho-syntactic structure of the sentences was not always obvious for the experimenter, CLUs were analyzed as whole units after transcription. The onset time and duration (in seconds) for every CLU was defined. Subsequently each CLU was assigned to one of the categories described separately for both experiments below, which capture morpho-syntactic symptoms appearing in continuous language production (see Figure 3).

To test the comparability of the healthy speakers' and the aphasic speech production, the following basic speech parameters were quantified with ASPA (Grande et al., 2008; Hussmann et al., 2012): percentage words, percentage open class words, type-token-ratio, syntactic completeness, complexity, MLU and words per minute. Differences between the aphasic speakers' output and the healthy speakers' picture descriptions in the 3W and NAT-condition were tested with the Mann-Whitney U test and between the 3W and the NAT-condition with the Wilcoxon signed rank test.

#### Experiment 1: categories for CLUs in the NAT and 3W-condition

When healthy participants described the pictures without limitation in the number of words (NAT), a CLU was classified as simple or complex, depending on whether or not it was part of a compound sentence. Beyond that each CLU was marked as complete or incomplete (see Grande et al., 2008 for details about criteria). The incompleteness was either caused by a missing verb or by a missing obligatory argument like an object.

In the 3-word condition (3W), a CLU was a simple sentence that was either complete or incomplete caused by a missing verb or by a missing obligatory argument like an object. Simple complete CLUs were further divided into morphologically correct or impaired CLUs, caused by at least one missing or wrong function word and/or inflectional element.

To capture the conceptual and morpho-syntactic planning of the CLUs (Grande et al., 2012) in all conditions pauses and interjections between two CLUs were classified as one category. The onset and duration of those periods were defined. Unintelligible or ambiguous CLUs were assigned to a separate category.

The resting periods between the pictures during which the participants saw a white screen served as a low-level baseline and were divided into mini-blocks of 25 s (see Maus et al., 2010 for the advantage of using short blocks in the range of 20 s).

#### Experiment 2: categories for aphasic speakers' CLUs

The aphasic participants' CLUs were assigned to one syntactical and one morphological category. On the syntactic level, utterances were classified just as in the healthy participants NAT-condition (complete, incomplete due to a missing verb, incomplete due to a missing obligatory argument).

On morphological level, morphological correct and impaired CLUs were distinguished. A CLU with an omission or substitution of a closed class word (e.g., a missing determiner, a wrong preposition) was classified in one category. When the error concerned the inflection of an open class word, the CLU was further assigned to a second morphological category. Because one CLU can contain morphological errors concerning open and closed class words at the same time, a mixed category was included. Morphological errors that were corrected were nonetheless assigned to one of the morphological categories.

### fMRI data analysis

The data were analyzed using SPM8 (Wellcome Department of Cognitive Neurology, UK) running on MATLAB 7.13 R2011b (The Mathworks Inc., Natick, USA) in combination with the SPM Anatomy Toolbox (Eickhoff et al., 2005) for the localization of effects. The data of all 15 healthy participants and three aphasic participants were pre-processed with the standard procedures of realignment to the mean image of the EPI time series using the Realign and Unwarp procedure provided in SPM8 in order to compensate for non-linear signal distortions potentially induced by head motion, normalization to the EPI template, spatial smoothing using a Gaussian kernel of 8 mm FWHM and a highpass filter of 1/128 Hz. Because the normalization procedure via unified segmentation failed in two aphasic participants, after the Realign and Unwarp and smoothing procedure, an alternative procedure was applied to the data of these two individuals, based on local non-linear transformations using registration with lesion masking (Hömke et al., 2009). The average head movement during scanning was small, mostly below the size of one voxel (Supplementary Material Table 1). To account for residual movement effects, the realignment parameters were included as regressors of no interest in the general linear model. One aphasic speaker (A4) showed a great spike in the resting period after the sixth picture. For this reason only data (behavioral and functional) up to the end of stimuli picture 6 (scan 346) were included into the study for A4.

Event-related data analysis was conducted by defining every uttered CLU as an event with its onset-time and duration. At the first (single subject) level, only conditions with at least 15 events were considered for statistical analysis (Grande et al., 2012). The onset vectors for each category were convolved with the canonical haemodynamic response function and its temporal derivative to account for minor latency differences (Friston et al., 1998).

#### Experiment 1: analysis on group level

For each participant, the contrasts of each category vs. the implicit resting baseline were calculated. The resulting contrasts were entered into two random-effects repeated-measures analyses at the second (group) level. Results on completeness, complexity, and morpho-syntactic planning were calculated in a second level analysis enclosing the following categories: complex complete CLUs, simple complete CLUs, pauses/interjections between CLUs in the NAT-condition as well as simple complete CLUs, simple incomplete CLUs with a missing verb, pauses/interjections between CLUs in the 3W-condition. The second level analysis to calculate results on the morphological processing contained the categories: simple complete CLUs with morphological errors, pauses/interjections between CLUs in the 3W-condition as well as simple complete CLUs and pauses/interjections between CLUs in the NAT-condition.

Specific effects were tested as linear t-contrasts. The calculated contrasts are listed in Table 2. Assuming that the differences between the processing of CLUs with different morpho-syntactic structures might be small, a Monte-Carlo simulation of the brain volume was employed to establish an appropriate voxel contiguity threshold (Slotnick et al., 2003). This correction has the advantage of higher sensitivity to smaller effect sizes, while still correcting for multiple comparisons across the whole brain volume (Sass et al., 2012). To correct for multiple comparisons at *P* < 0.05, a cluster extent of 14 contiguous resampled voxels was indicated as necessary in the control group assuming an individual voxel Type I error of *P* < 0.001.

**Table 2:** **Calculated t-contrasts for the different morpho-syntactic aspects**.

	**fMRI contrasts**	
	**Exp. 1: healthy speakers**	**Exp. 2: agrammatic speakers**
Completeness	Simple complete CLUs in the 3W-condition vs. simple CLUs that are incomplete due to a missing verb in the 3W-condition	Simple complete CLUs vs. simple CLUs that are incomplete due to a missing verb
Complexity	Complex complete CLUs in the NAT-condition vs. simple complete CLUs in the NAT-condition	Only A1: complex complete CLUs vs. simple complete CLUs
Morphology	Simple complete CLUs in the NAT-condition vs. simple complete CLUs with morphological errors in the 3W-condition	Morphologically correct CLUs vs. CLUs with omission and/or substitution of closed class words
Morpho-syntactic planning	Pauses/interjections between CLUs in the 3W-condition vs. pauses/interjections between CLUs in the NAT-condition	No comparable contrast

*Exp., experiment; CLU, clause-like unit; A1, patient A1*.

#### Experiment 2: analysis on single subject level

The aphasic participants' data were analyzed on single subject level in order to avoid problems associated with grouping data from patients with heterogeneous lesion sizes and locations and with small sample sizes (Price et al., 2006). By always assigning the CLUs of the aphasic speakers to two categories, results for syntax and morphology were calculated in two analyses. The first level analysis to calculate results on completeness, complexity and morpho-syntactic planning and the first level analysis to calculate morphological processing enclosed the particular categories on the concerning aspects.

Specific effects were tested as linear t-contrasts (see Table 2 for the calculated contrasts). To correct for multiple comparisons at *P* < 0.05 in the aphasic speakers, a cluster extent of 27 contiguous resampled voxels was indicated as necessary assuming an individual voxel Type I error of *P* < 0.01 (Monte-Carlo simulation).

To find out if the brain regions showing a significant BOLD effect in the healthy speakers were involved in the processing of the same morpho-syntactic aspects in the aphasic speakers, the spmT-values at the healthy speakers' local maxima (voxels with activation peaks in the clusters of activity) of the t-contrasts corrected at cluster level (*P* < 0.05, to reduce the number of maxima) were extracted. SpmT-values indicate the intensity of activation at the given voxel. In the correspondent aphasic speakers' t-contrasts the spmT-values were extracted at the equivalents of the groups' local maxima and compared to the healthy speakers' maxima with a one-sample *t*-test.

## Results

### Experiments 1 and 2: behavioral data

When describing pictures without a limitation of words, the healthy participants produced an average of 32.9 CLUs per picture with a MLU of 6.6 words. In the 3W-condition (MLU: 3.1) the average number of CLUs per picture reduced to 24.4. The aphasic speakers' performance is shown in Table 3. Information on the morpho-syntactic classification of all CLUs is shown in Table 4. Only A1 produced enough complex complete sentences and A2, A3, and A5 enough CLUs with incorrect inflection of open class words to analyze the correspondent functional data. Because the latter could not be analyzed in the healthy speakers, these results will not be included in this paper. The morphological category with omissions and/or substitutions of closed class words comprised almost entirely CLUs with omissions of function words in the healthy speakers' 3W-condition. The aphasic speakers showed different performances concerning the production of function words: A2 presented only with omissions, A1 and A4 produced CLUs with omissions and substitutions and A3 and A5 showed primarily substitutions.

**Table 3:** **Average amount of CLUs per picture and MLU in the aphasic participants**.

	**A1**	**A2**	**A3**	**A4**	**A5**
CLUs/P	15.1	17	23.4	16.5	16.7
MLU	6.5	4.5	6.5	3.7	5.1

*CLUs/P, clause-like units per picture; MLU, mean length of utterance*.

**Table 4:** **Occurrence of the assigned categories related to the total number of CLUs in percent**.

	**NAT**	**3W**	**A1**	**A2**	**A3**	**A4**	**A5**
Simple-complete	51.9	7.0	53.3	40.0	49.1	24.7	39.8
Simple-incomplete-missing verb	5.2	47.3	13.9	34.0	20.6	28.9	45.5
Simple-incomplete-missing argument	2.7	8.2	11.7	26.0	21.5	43.3	12.2
Complex-complete	36.8	Not assigned	16.8	0.0	6.1	3.1	5.7
Complex-incomplete-missing verb	2.1	Not assigned	2.9	0.0	1.9	0.0	2.4
Complex-incomplete-missing argument	1.3	Not assigned	1.5	0.0	0.9	0.0	1.6
Pauses/interjections between CLUs	51.5	90.4	75.2	62.0	65.9	83.5	76.4
Unimpaired	Not assigned	7.0	61.3	22.7	40.2	42.3	51.2
Morphological errors-omissions and substitutions of closed class words	Not assigned	37.5	32.1	42.0	43.9	43.3	37.4
Morphological errors-missing/wrong inflection of open class words	Not assigned		4.4	12.7	9.3	6.2	10.6
Morphological errors-mixed	Not assigned		2.2	22.7	6.5	8.2	8.1

*NAT and 3W: mean values; CLU, clause-like unit*.

The statistical analysis of differences between the aphasic speakers' and the healthy participants' speech production in the 3W and NAT-condition revealed significantly lower values in the aphasic speakers' group (AS) and in 3W as in NAT for completeness (AS < NAT: *P* = 0.001; 3W < NAT: *P* = 0.001) and in the speech rate (words per minute) (AS < NAT: *P* = 0.002; 3W < NAT: *P* = 0.001). The complexity was lowest in 3W and highest in NAT with significant differences between the three groups (3W < AS: *P* = 0.001; 3W < NAT: *P* < 0.001; AS < NAT: *P* = 0.001). The aphasic speakers showed a significantly lower percentage of words than the healthy speakers in both conditions (AS < 3W: *P* = 0.006; AS < NAT: *P* = 0.014) as well as a lower type token ratio (AS < 3W: *P* = 0.005; AS < NAT: *P* = 0.001). The percentage of open class words was significantly higher in 3W than in NAT and in the aphasic speakers (AS < 3W: *P* = 0.001; NAT < 3W: *P* = 0.001).

### Experiment 1: functional data of healthy speakers

#### Completeness

**Figure 4:**
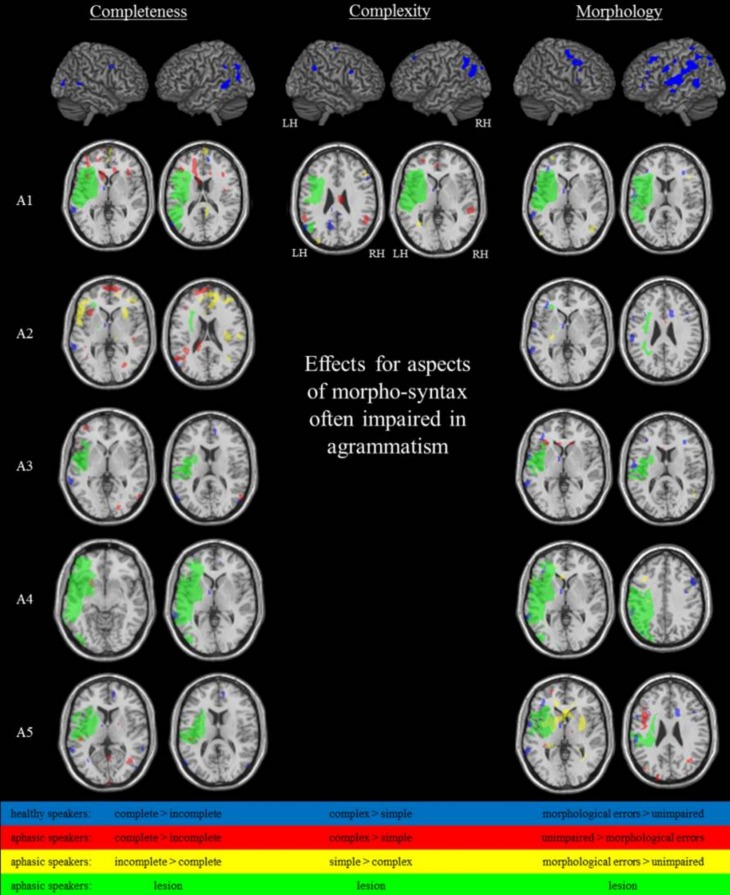
**Brain activation during the spontaneous language task at *P* < 0.001_uncorr_, extend threshold *k* ≥ 14 voxels (healthy speakers) or *P* < 0.01_uncorr_, extend threshold *k* ≥ 27 voxels (aphasic speakers/A1)**.

Contrasting all CLUs classified as simple and complete against all simple and incomplete CLUs that were missing a verb (each 3W-condition) revealed an effect in the left pMTG extending to the angular gyrus (AG) in the inferior parietal cortex. In addition, a homologous cluster with a maximum in the pMTG showed that was smaller in extent though. Further activation clusters were found in the left hemisphere in the precuneus, thalamus, the paracentral lobule, and the inferior parietal lobule (IPL). In the right hemisphere effects showed in the anterior cingulate cortex, the middle occipital gyrus and the right MFG. See Figure 4 and Supplementary Material Table 2 for details.

There was no BOLD effect found for the reverse contrast contrasting incomplete with complete simple CLUs.

#### Complexity

An effect for complexity, i.e., the contrast of complex with simple CLUs (both complete and NAT-condition, Figure 4 and Supplementary Material Table 3), was found in the left and right middle cingulate cortex, the postcentral gyrus, the AG, the left pMTG, and the IPL. Further activation clusters showed in the left cuneus and precuneus and the thalamus. Right hemisphere clusters were found in the supramarginal gyrus (SMG) and the pars opercularis of the IFG. Bilateral effects were located in the middle occipital and superior frontal gyrus.

Again the reverse contrast did not result in any significant activation cluster.

#### Morphology: omission of function words

In the 3W-condition the morphological errors in simple complete CLUs comprised almost solely the omission of function words. The contrast of all CLUs classified as simple and complete in the NAT-condition with those CLUs assigned to be simple complete but with the omission of function words in the 3W-condition revealed no BOLD differences.

An effect for the omission of function words in the reverse contrast showed in a widespread network of activation. The largest cluster comprised the SMG, the Rolandic operculum and the IPL in the left hemisphere. The strongest effects showed in the left pMTG, the right postcentral gyrus, and the anterior cingulate cortex. Further left hemisphere clusters were located in the pars triangularis of the IFG, the anterior MTG, the pre- and postcentral gyrus, and the thalamus. Bilateral BOLD effects showed in the pars opercularis of the IFG, the Rolandic operculum and the MFG. See Figure 4 and Supplementary Material Table 4 for detailed information on the results.

#### Morpho-syntactic planning

The contrast of pauses and interjections between CLUs in the NAT-condition with those in the 3W-condition revealed one left hemispheric cluster with increased activation in the left pMTG. In the right hemisphere effects showed in the AG and SMG, the precuneus and the insula.

An effect for the morpho-syntactic planning in the 3W-condition in the reverse contrast was found in the left pars triangularis and bilaterally in the pars opercularis of the IFG. Further frontal activation clusters in the left hemisphere appeared in the insula lobe, the precentral gyrus, and the superior frontal gyrus. In the left MTG and the right cerebellum additional effects were found. See Supplementary Material Table 5 for detailed information on the results.

### Experiment 2: functional data of aphasic speakers

#### Comparison of spmT-values: completeness and complexity

The comparison of the spmT-values (indicating intensity and significance of activity) of the individual aphasic speakers with the spmT-values of the healthy group at the nine local maxima (i.e., voxels with activation peaks within the cluster of activity above the chosen level of significance) for completeness reported in experiment 1 (corrected on cluster level, *P* < 0.05) revealed deviant activation strength at at least four maxima in all aphasic speakers except A4 (one-sample *t*-test, *P* < 0.05). In A1 and A3 deviant spmT-values were significantly lower, in A2 and A4 either significantly higher or lower than the healthy speakers' values. All maxima were affected. The spmT-value at the maximum in the IPL was significantly lower in two and significantly higher in the other two aphasic speakers. A4 had a lesion at six of the nine maxima. See Table 5 for details.

**Table 5:** **Comparison of aphasic speakers' spmT-values and the groups' mean at the coordinates of the healthy speakers' maxima**.

**Local maximum in macroanatomical structure**	**MNI coordinates**			**A1**	**A2**	**A3**	**A4**	**A5**
	***x***	***y***	***z***					
**SIMPLE-COMPLETE > SIMPLE-INCOMPLETE-MISSING VERB**								
L middle temporal gyrus	−62	−42	4	▼	▲		Lesion	
L middle temporal gyrus	−58	−70	14			▼		▼
L angular gyrus	−54	−70	38		▼	No value	No value	▼
L middle temporal gyrus	−60	−50	10	Lesion		▲	Lesion	
L middle temporal gyrus	−56	−68	26			▼		▼
L inferior parietal lobule	−56	−42	36	▼	▲	▼	Lesion	▼
L angular gyrus	−56	−58	28	▼	▲		Lesion	▼
L middle occipital gyrus	−44	−76	24	Lesion	▲	▼	Lesion	▼
L middle temporal gyrus	−58	−52	−2	▼			Lesion	
**COMPLEX-COMPLETE > SIMPLE-COMPLETE**								
L postcentral gyrus	−24	−36	58					
L cuneus	−10	−64	26	▲				
L middle cingulate cortex	0	−26	36					
L middle cingulate cortex	−10	−4	34	▼				
L posterior cingulate cortex	−8	−34	30					
L lingual gyrus	−12	−84	−6	▼				
L middle cingulate cortex	−6	−40	38	▲				
R middle cingulate cortex	4	−2	44	▼				
L posterior cingulate cortex	−4	−38	32	▲				
L precuneus	−8	−44	64	▲				
L precuneus	−24	−38	42	▼				
L angular gyrus	−54	−70	32	▼				
L middle temporal gyrus	−54	−62	22					
L inferior parietal lobule	−50	−54	46	▲				
L middle occipital gyrus	−38	−72	36	▼				
L inferior parietal lobule	−54	−48	36	▲				
L inferior parietal lobule	−56	−52	40					
L supramarginal gyrus	−58	−30	28	▼				

*▼, significant lower spmT-value; ▲, significant higher spmT-value; one-sample t-test, P < 0.05*.

The spmT-values for complexity at the local maxima of experiment 1 could only be compared between A1 and the healthy speakers. A1 presented with seven significant lower and six significant higher spmT-values (out of 18 maxima in two clusters, Table 5).

Because the morphological category concerning closed class words comprised different symptoms (omissions and/or substitutions of function words) in the group of healthy speakers and the aphasic speakers, the spmT-values of this contrast were not compared.

#### Completeness

Results of aphasic speakers' fMRI data for the contrast of simple complete vs. simple incomplete CLUs with a missing verb are graphically presented in Figure 4. See Table 6 for detailed information on the areas activated in the individual aphasic speakers. Analyses revealed an effect for complete CLUs in the left frontal lobe in A1, A2, A3, and A5, in temporal areas of the left and right hemisphere in A2, A3 (only LH), and A5, and in the parietal cortex in A2 (LH) and A5 (RH). The precuneus and the cingulate cortex were activated in A3. A4 showed hardly any significant differences in activation patterns.

**Table 6:** **Aphasic speakers showing significant fMRI activation differences by region and contrast at *P* < 0.01_uncorr_, extend threshold *k* ≥ 27 voxels based on Monte Carlo correction**.

	**Completeness**				**Complexity**				**Morphology**			
	**Simple-complete > simple-incomplete-missing verb**		**Simple-incomplete-missing verb > simple complete**		**Complex-complete > simple-complete**		**Simple-complete > complex-complete**		**Unimpaired > morph. errors-closed class words**		**Morph. errors-closed class words > unimpaired**	
**Area**	**LH**	**RH**	**LH**	**RH**	**LH**	**RH**	**LH**	**RH**	**LH**	**RH**	**LH**	**RH**
IFG _(p. operc.)_	A2										A5	
IFG _(p. triang.)_	A1	A1	A2					A1	A4	A1	A3	
	A2										A5	
IFG _(p. orb.)_		A1	A2				A1				A3	
RolOp	A5			A2								
MFG	A1	A1	A2	A2			A1		A4		A3	
	A2											
	A3											
SFG	A1	A1		A1					A1		A5	A5
	A2	A2		A2								
	A3			A3								
	A4											
PrecG	A1			A2			A1				A5	
	A2											
PostcG		A5		A2					A4			
Insula lobe	A2	A1							A5	A5		
STG	A5	A1	A2	A2							A5	A5
		A2										
MTG	A2	A2		A1	A1	A1	A1			A1	A5	
		A3								A3		
		A5										
ITG	A2	A3		A2			A1		A1			
		A5										
Temporal pole		A1	A3								A3	
											A5	
AG	A2			A1		A1					A5	
				A2								
SMG		A5	A1	A1	A1							
			A2	A2								
IPL	A2			A1					A2			
SPL	A2	A1							A2		A5	
Precuneus	A3	A3	A1	A1	A1	A1	A1		A1			A3
			A2						A3			A5
Thalamus	A1	A3		A1	A1				A2	A5		A5
	A5											
Hippocampus									A5			
Cuneus		A3				A1				A1		
Cingulate cortex	A3		A1	A1	A1	A1			A1		A2	A5
			A4	A2					A4			
			A5						A5			
Occipital cortex	A1	A1			A1	A1	A1		A1	A1	A5	A5
	A2	A2							A2	A5		
	A5	A3							A4			
		A4										
		A5										
Cerebellum	A2	A1	A1		A1		A1			A4	A3	A3
		A2								A5		
		A3										
		A5										

*LH, left hemisphere; RH, right hemisphere; SFG, superior frontal gyrus; PrecG, precentral gyrus; PostcG, postcentral gyrus*.

An increased activation pattern for incomplete utterances with a missing verb was found in left and right frontal and in right temporal areas in A2. In A1 and A2 effects were found in left and right parietal regions. Effects in the cingulate cortex showed in A1, A2, A4, and A5, in the precuneus in A1 and A2. Overall, smaller differences in activation patterns were found in A3, A4, and A5.

#### Complexity

Only A1 showed a sufficient amount of CLUs that were complex and complete (Table 4) to analyze incidental functional data in the contrast with simple complete CLUs (Figure 4). An effect for complexity was found in the MTG in both hemispheres, the left SMG and the right AG and bilaterally in the occipital cortex, the cingulate cortex and the precuneus.

The reverse contrast resulted in an effect for simple sentences in the left and right frontal cortex, the left temporal and occipital cortex and the precuneus.

#### Morphology: omission and/or substitution of function words

Figure 4 shows the results for the contrast of morphologically correct CLUs vs. CLUs with omissions and/or substitutions of closed class words. Detailed information on the areas activated in the individual aphasic speakers is given in Table 6. An effect for morphologically correct CLUs appeared in the frontal lobe in A1 (LH, RH) and A4 (LH). Temporal activation showed in A1 (LH, RH) and A3 (RH). Clusters in the parietal lobe were only found in A2. Increased activation in the bilateral insula lobe showed in A5. A1, A4, and A5 showed an effect in the cingulate cortex, A1 and A3 in the precuneus.

When compared with morphologically correct CLUs, BOLD effects for morphological errors were found in frontal and temporal regions in A3 (LH) and A5 (LH, RH), who both primarily presented with substitutions of function words. Left hemispheric parietal activation appeared in A5. Additional clusters included the cingulate cortex in A2 (only omissions of function words) and A5 and the precuneus in A3 and A5. There was no BOLD effect found for A1 and A4 in this contrast, who both showed CLUs with omissions as well as substitutions of closed class words. A3 showed distinctly more effects for the morphological errors than in the reverse contrast for the correct CLUs.

## Discussion

In the present fMRI study, the neural correlates of syntactic completeness, complexity and the production of function words in normal and disordered grammatical processing in continuous language production were studied with a new paradigm. In experiment 1, the neural mechanisms underlying natural morpho-syntactic processing in a picture description task were analyzed in 15 healthy speakers. In the same group, agrammatic-like speech behavior was induced to study the underlying functional processes. In experiment 2, five agrammatic participants performed the picture description task to gain insights in the neural correlates of agrammatism and the functional reorganization of language processing after stroke. In both experiments, agrammatic language production was contrasted with unimpaired language production. The main results shall now be discussed in detail.

### Experiment 1: natural vs. artificially reduced morpho-syntax in healthy speakers

In experiment 1, non-brain-damaged participants described pictures with natural spontaneous speech on the one hand and with utterances with a reduced morpho-syntax (at most three words per utterance) on the other hand. By contrasting corresponding categories we were able to reveal those brain regions underlying three aspects of spontaneous speech production that are often impaired in agrammatism: syntactic completeness, complexity and the correct use of function words. All aspects were associated with activation in the left pMTG and the IPL. Complex and complete CLUs were associated with an additional effect in the left AG and SMG. The omission of function words was linked to an additional bilateral frontal effect in the pars triangularis and pars opercularis of the IFG, the Rolandic operculum and the MFG. These findings are in line with earlier studies on morpho-syntactic processing and give further insights into the neural mechanisms underlying spontaneous language production.

Studies on sentence production reported stronger activation for syntactically more elaborated conditions, e.g., complete vs. incomplete CLUs (Grande et al., 2012) or CLUs consisting of sentences vs. noun phrases vs. syntactically unrelated words (Indefrey et al., 2001a, 2004). Here, the results suggest a similar effect for the production of complete and complex sentences implying greater morpho-syntactic demands than incomplete and simple sentences. An omission of a verb implies no processing for verbal inflection and agreement and thus reduced effects in areas associated with these processes. The phrasal architecture of simple sentences consists of less syntactic layers und thus less syntactic integration processes as in syntactically complex hierarchically structured compound sentences (Bornkessel et al., 2005). The brain regions associated with the production of CLUs with increased morpho-syntactic demands in our study fit earlier reports on different aspects of morpho-syntactic processing in healthy speakers that mostly involved receptive language processing tasks on phrase or sentence level (Meltzer et al., 2010; Tyler et al., 2010, 2011). Shetreet et al. (2009) described activation in the left IFG, bilateral temporo-parietal cortices, bilateral MTG, and precuneus when contrasting the processing of heard embedded vs. simple sentences. Thompson et al. (2007, 2010a) reported an engagement of the SMG and AG to be associated with increased argument structure elaboration. Here, the production of embedded CLUs in spontaneous speech was associated with an effect in the same temporal and parietal regions. The results suggest that the processing of structures with increased morpho-syntactic elaborateness in language production involves a resembling network of temporo-parietal areas than the earlier described network for language reception. In a picture description task, Kircher et al. (2005) also found the activation in the left MTG to be positively correlated with the number of complex, i.e., compound sentences produced. We could confirm further inferior parietal areas besides the pMTG to be involved in the production of complex sentence constructions in spontaneous speech production. The relevance of the temporal and parietal areas for the production of complex and complete sentences is also reflected in the conceptual and morpho-syntactic planning (pauses/interjections between CLUs) in the NAT-condition when contrasted with planning in the 3W-condition with one variation: unlike during production of the CLUs, the activation peaks were found in the AG and SMG of the right instead of the left hemisphere during planning. Against our expectation and the literature on sentence comprehension (e.g., Shetreet et al., 2009) and production (e.g., Indefrey et al., 2001a, 2004), activation in the IFG and the Rolandic operculum were not associated with completeness or complexity in spontaneous language production. Instead, those areas seemed to play a decisive role in the encoding and conscious processing of closed class words in sentence production, as will be discussed below.

The production of a complete CLU with a verb or of a complex sentence construction with CLUs in compound sentences is not only associated with greater morpho-syntactic demands but also requires more semantic encoding and lexical retrieval. In complete CLUs the verb and if required its obligatory arguments are retrieved. Agrammatic speakers often show a disproportionate verb processing disorder as compared to noun processing. Those deficits are assumed to reflect not only syntactic but also lexical and semantic dysfunctions (Mätzig et al., 2009; Luzzatti et al., 2012). In complex sentences the thematic concepts and lexical entries that are retrieved are linked to a logical chain more complex than in a simple sentence with regard to content and argument structure. Activation peaks in the pMTG and STG, in the AG and SMG that we found for completeness and complexity have been related to lexical retrieval and semantic processes in several studies (Indefrey and Levelt, 2004; Vigneau et al., 2006; Indefrey, 2011). The possibility that our results for completeness and complexity do as well reflect semantic and lexical processes thus cannot be entirely excluded. However, by contrasting always two categories that differed only in one syntactic feature [complex complete CLUs vs. simple complete CLUs, complete simple CLUs vs. incomplete (missing verb) simple CLUs] only the specific effects associated with completeness or complexity in natural or reduced morpho-syntax were captured.

In the 3W-condition participants had to reduce their utterance length to a maximum of three words. The omission of closed class words was one way to accomplish this. Those omissions were associated with activation in a network including again the left pMTG and the IPL. Notable are particularly the effects in the left frontal areas that showed bilaterally but stronger in the left hemisphere. Activation peaks in the IFG and MFG have often been associated with morpho-syntactic encoding like the processing of grammatical gender, gender violations and syntactic and morphological error detection in production and comprehension (Embick et al., 2000; Indefrey et al., 2001a, 2004; Moro et al., 2001; Miceli et al., 2002; Friederici et al., 2003; Heim, 2008; Heim et al., 2010). A possible explanation for effects in these areas although function words were omitted might be an intentional suppression of the closed class words after their syntactic encoding to artificially reduce the utterance length. The contrast of the two morpho-syntactic planning-conditions corroborates the assumption that conscious morpho-syntactic processing is associated with frontal activation: For morpho-syntactic planning in the 3W-condition effects were found in the left pars triangularis and bilateral pars opercularis of the IFG, although the syntactically more elaborated sentences were planned in the NAT-condition. In the 3W-condition the frequency and the duration of pauses in between CLUs were considerably higher than in the NAT-condition. This is in favor of an intentional planning and morpho-syntactic processing in the 3W-condition. In sum, the inferior and middle frontal cortex seemed to be involved in the encoding and conscious processing of closed class words in sentence production.

In experiment 1, all analyzed contrasts revealed activation in prefrontal regions including the cingulate cortex, that have been associated with general performance and speech monitoring (Christoffels et al., 2007; Price, 2010). The contrasts for the omission of function words and the morpho-syntactic planning in the 3W-condition showed supplementary effects in the precentral gyrus, the right cerebellum, the bilateral SMA and the left insula lobe which have all been related to monitoring functions (e.g., pre-error monitoring) in language processing (Ullsperger and Von Cramon, 2004; Christoffels et al., 2007; Abel et al., 2009; Price, 2010). These results support the assumption that both categories were associated with increased attention and error-monitoring processes as agrammatic-like CLUs had to be produced.

In summary, reduced morpho-syntactic demands in incomplete sentences and sentences without embedding were associated with reduced effects in the pMTG and the inferior parietal cortex including the AG and SMG mainly in the left hemisphere. Morphological errors in terms of omissions of function words and the intentional manipulation of morpho-syntactic structures in CLUs were associated with activation peaks in the pMTG, the parietal lobule, the IFG, MFG, and areas subserving monitoring processes. These findings may serve as the basis for interpreting the agrammatic speakers' results.

### Experiment 2: agrammatic morpho-syntax in aphasic speakers

If the results in experiment 1 suggest that agrammatic-like symptoms were artificially induced in healthy speakers, the activation networks discussed above should also play a role in sentence production in the five agrammatic participants (experiment 2). For this reason the intensity of activity at the healthy speakers' activation peaks (indicated by the spmT-values extracted at the local maxima) and the intensity of activity (spmT-values) in the aphasic speakers at the equivalent voxels of the groups' local maxima were compared. In every agrammatic speaker (except A4) and each contrast, at least four equivalents of the groups' local maxima showed deviant activation strength which was significantly increased at some and decreased at other maxima. Regarding the maxima that showed divergences the five aphasic speakers did not present with consistent activation patterns. One maximum could be associated with increased activation in the one and with decreased activation in the other aphasic speaker. Critically one can note that the healthy speakers' categories varied from the aphasic speakers' categories and thus the contrasts were not comparable. This is, to a certain amount, true for the 3W-categories used in the completeness contrast that were compared with the aphasic speakers' categories not underlying any limitations in utterance length. However, in both groups the contrasted categories differed only in one feature: the verb that was produced or not. In the healthy and the aphasic speakers the fMRI results for this contrast thus revealed the areas associated with completeness (verb production) in sentence generation. In the second comparison (complexity), the two contrasted categories contained only CLUs of the NAT-condition, where speakers described the pictures without a target utterance length just like the aphasic speakers did. Still the comparison of A1's spmT-values with that of the healthy speakers' revealed increased and decreased deviations at several maxima. In sum, the aphasic speakers showed individual activation patterns at the local maxima of the healthy speakers with deviant activation strength at atleast four maxima per contrast.

This analysis suggests that the basis of morpho-syntactic processing in the lesioned brain varies from that in healthy speakers. To get further insight into the individual activation patterns, we contrasted brain activation correlated with agrammatic CLUs with activation of undisturbed speech production in the aphasic speakers. As in experiment 1, the three aspects of spontaneous speech production often impaired in agrammatism (syntactic completeness, complexity and the correct use of closed class words) were analyzed.

In experiment 1, incomplete CLUs with a missing verb were associated with decreased activation. In experiment 2, two aphasic participants (A3 and A5) showed a similar activation pattern but with additional activation in areas not specifically involved in language processing. In two aphasic speakers (A1 and A2) incompleteness was associated with increased as well as decreased activation. In A4 differences in both directions did not show in language-specific areas. Only A1 produced enough complex complete CLUs to analyze the effects in activation between complex (with embedding) and simple CLUs. The production of simple sentence constructions was associated with decreased activation when compared with complex sentences in the group of healthy speakers. A1 showed the same pattern but with additional increased activation. The category morphological errors concerning open class words embraced different symptoms in the individual aphasic speakers: While A1 and A4 produced omissions and substitutions of function words, A2 showed primarily omissions (like the healthy speakers) and A3 and A5 predominantly substitutions of function words. Omissions of function words were associated with increased activation in the healthy speakers but with primarily decreased activation in the aphasic speakers as was also found for the combination of omissions and substitutions. Substitutions however, were correlated with primarily increased activation in language specific areas. Thus, neither for the different symptoms nor for the individual aphasic speakers stringent activation patterns (decreased and/or increased) were found. In the literature aphasic errors have mostly been associated with an enhancement in activation in right and left hemisphere regions subserving language and monitoring processes (Fridriksson et al., 2009; Postman-Caucheteux et al., 2010; Tillmanns et al., 2011). Different explanations for enhanced activation during aphasic errors can be found. They were ascribed to increased language specific processing as well as monitoring and control processes to overcome the troubles in speech production (e.g., in word finding difficulties, Tillmanns et al., 2011) or to an increased activation (especially in the right hemisphere) causing speech errors (e.g., Naeser et al., 2004; Postman-Caucheteux et al., 2010). Studies with healthy speakers report besides enhanced effects also reduced activation for speech errors or less elaborated morpho-syntactic structures as were found in our data (Indefrey et al., 2004; Abel et al., 2009; Grande et al., 2012). As discussed earlier, experiment 1 revealed reduced activation for simple and incomplete utterances while omissions of function words were associated with enhanced activation. In experiment 2 the results suggest that agrammatic errors in spontaneous speech are associated with an individual pattern of decreased and enhanced activation in left and right hemisphere regions when contrasted with unimpaired language production and language production in the healthy speakers.

The role of both hemispheres in the functional reorganization process in aphasia has been discussed controversially. Activation in preserved and perilesional task-specific regions in the lesioned language dominant hemisphere are attributed an important role in the functional reorganization and recovery process (Rosen et al., 2000; Heiss et al., 2003; Fernandez et al., 2004; Saur et al., 2006; Meinzer et al., 2008; Tyler et al., 2010). Here, the analysis revealed effects in preserved and perilesional areas that were always accompanied by activation in the non-lesioned hemisphere. Some salient results shall be discussed.

Although some left hemisphere areas were not directly affected by the lesion, they showed deviant activation strength. One example is the comparison of spmT-values between the healthy speakers and A2, who presented with a subcortical lesion and yet showed deviant spmT-values in left hemisphere task-specific regions. This might be due to a diaschisis effect, a disruption in the connections between the lesion and language relevant areas resulting in aphasic speech (Price et al., 2001). The spmT-values in perilesional maxima appeared to be without a significant difference or significantly decreased when compared with the healthy speakers' values. But the results of the functional data revealed partly significantly increased BOLD effects in perilesional tissue. This confirms earlier results that besides preserved left hemisphere language specific regions and right hemisphere homolog areas some (but not all) perilesional tissue might be capable of taking over functions of the lesioned brain regions (Cao et al., 1999; Rosen et al., 2000; Zahn et al., 2004; Saur et al., 2006). It should be noticed that the lesions of the five aphasic speakers differed in size and location by which the contemplable perilesional tissue for reintegration varied (Thompson and den Ouden, 2008).

In the aphasic speakers, the aspect of completeness revealed enhanced activation in bilateral frontal, temporal and parietal regions unlike the healthy speakers' effects that showed mainly in left temporal and parietal areas. Coactivation and compensational activation in homolog areas have often been associated with facilitating functions (Heiss et al., 1999; Thulborn et al., 1999; Abo et al., 2004; Meinzer et al., 2006, 2012; Elkana et al., 2011) but also with constraint mechanisms (Blank et al., 2003; Hamilton et al., 2010; Postman-Caucheteux et al., 2010; Szaflarski et al., 2013). Naeser et al. (2004) described an increased activation in right hemisphere areas in non-fluent speech production of aphasic speakers when compared with healthy speakers. The authors interpreted this activation as dysfunctional processes causing hesitant speech rather than reflecting compensatory activation. Evidence for the fact that the right hemisphere does not play a crucial role in the recovery of syntactic processing was reported by Tyler et al. (2010) who did not find any correlation between right hemisphere activity and damage in the left hemisphere or with performance in receptive syntactic processing. In our study on syntax production, right hemispheric activation went along with agrammatic as well as unimpaired speech production. Bilateral activation peaks in the IFG were associated with complete CLUs in A1. At the same time A1 showed effects in the right AG and IPL when producing incomplete CLUs, homolog areas of those associated with completeness in healthy speakers. Several studies have obtained improved language functions by inhibiting the functions of the right pars triangularis via TMS (Hamilton et al., 2010; Naeser et al., 2011). Here, the activity in this area was significantly increased when simple sentences were contrasted with complex sentence constructions in A1. In line with earlier results, a relatively higher activation in the right pars triangularis might thus have a negative impact on the production of sentences with embedding. However, more aphasic speakers' data on this effect would be necessary to draw any conclusion. And also the reversed effect showed, disagreeing earlier results: the right pars triangularis was associated with decreased activation for incomplete CLUs and morphological errors. Right hemispheric activation might thus improve recovery, but some regions might be less efficient than the lesioned areas, resulting in agrammatic speech production (although it should be noted that all effects were always associated with activation in networks of brain regions and not just one specific region). Other regions might inhibit the recovery by interhemispheric inhibition (Hamilton et al., 2010; Turkeltaub et al., 2011). These mechanisms seem to differ among the individual lesioned brains. In summary, activation showed in preserved and perilesional areas in the left hemisphere, which might be intrahemispheric compensations by recruiting brain areas next to the lesion that are normally not employed for the specific language function. Additional right hemisphere activation might be interhemispheric compensational processes of dysfunctional areas directly or indirectly affected by the lesion (Heiss et al., 2003; Price and Crinion, 2005) or dysfunctional processes in terms of increased activation. Overall the aphasic speakers in this study showed stronger bilateral BOLD responses than the healthy speakers.

Aphasic speakers recruited neural tissue beyond the network supporting the specific morpho-syntactic processes in healthy speakers (experiment 1) that has, however, been reported to be involved in syntactic or general language processing, monitoring and attention processes (Vigneau et al., 2006; Price, 2010; Indefrey, 2011; Vigneau et al., 2011). While the inferior frontal areas seemed to play a crucial role in the healthy speakers only in the conscious processing of morphology, all aphasic speakers except A4 showed effects in these areas for completeness as well (and complexity in A1). This is in line with earlier studies on morpho-syntactic aspects of speech production (Haller et al., 2005; Shetreet et al., 2009; Segaert et al., 2011). Perhaps the inferior frontal areas, which play a role in morpho-syntax, have the potential to take over these specific functions in functional reorganization, even though they do normally not account for the difference between complete and incomplete CLUs (but see Grande et al., 2012 for a contradictory result). Another explanation could be increased morpho-syntactic processing requirements in agrammatic speakers that also emerged in the healthy speakers' 3W-condition. In line with that did all aphasic speakers reveal effects much more pronounced than the healthy speakers did in areas related to monitoring, control, and attention processes and not specifically to language functions. This indicates that morpho-syntax demands stronger processing capacities in agrammatic speakers.

The activation patterns in the agrammatic speakers seemed to be strongly influenced by lesion size and location and related to the participants' language capacities. That agrammatism is associated with lesions in various locations that go beyond Broca's area (Cappa, 2012) is in line with lesion sizes and locations in our study that differed between the aphasic speakers. A lesion overlap revealed that all five lesions overlapped only in part of the insula lobe and the putamen, close to activation associated with intentional planning and morpho-syntactic processing of CLUs with a reduced morpho-syntactic structure in the 3W-condition in experiment 1. These areas have been related to performance monitoring and control processes in speech processing (e.g., Ullsperger and Von Cramon, 2004; Christoffels et al., 2007; Ketteler et al., 2008). A lesion here might have a negative impact on the aphasic speakers' capacities to compensate their language deficit resulting in agrammatic speech production. In all aphasic participants except A2 further areas fully or partly affected by the lesion included areas 44 and 45 in the left IFG, the parietal operculum, the superior temporal and the inferior parietal cortex. In sum the lesion sites and sizes were heterogeneous in the five aphasic speakers which might have an impact on the divergent severity of aphasia and agrammatic symptoms. The statistical analysis of differences between the group of five aphasic speakers' and the group of healthy speakers' speech production in the 3W and NAT-condition revealed, that the aphasic speakers did not present with a severe deficit in their syntactic language functions as they approached the values of the NAT-condition although they all had been classified with a spontaneous language syntax scale score 2 on the AAT (Huber et al., 1983). The percentage of open class words was significantly lower than in the 3W-condition. This result reveals that the aphasic speakers did use function words, unlike many aphasic speakers with severe agrammatism (De Roo et al., 2003; Milman et al., 2008). However, the data do not convey any information about the correctness of the closed class words used. In fact four of the five aphasic participants (A1, A3, A4, and A5) showed many substitutions of function words and A2 was the only aphasic speaker with only omissions of function words. As discussed earlier this heterogeneity is also reflected by the functional data. The amount of complex CLUs was in between the 3W and the NAT-conditions. The completeness and the speech rate of the aphasic speakers resembled the values of the healthy speakers in the 3W-condition. Thus, both groups left out verbs or syntactically necessary elements of the sentence and showed a hesitant speech with pauses for sentence planning. Word-finding difficulties presenting in all five aphasic participants had an impact on the speech rate, the percentage of words and word variability (type-token-ratio), the latter two were significantly lower than both conditions in the healthy speakers. Hesketh and Bishop (1996) reported a corrective adaptation of aphasic speakers in a picture description task. The present results reveal that this could have applied to the aphasic speakers in this study as well what might explain the divergence between classification and behavioral data. In that case they would have quitted their adaptive strategy (short CLUs with reduced morpho-syntactic structure) and produced longer but morpho-syntactically incorrect and non-fluent utterances. The classification of the aphasic speakers' CLUs in the morpho-syntactic categories under study revealed the individual differences in type and severity of agrammatic symptoms. These heterogeneous language patterns are reflected by the results of the functional data. Thus, besides the lesion size and location, the remaining language capacities and strategies of aphasic speakers to compensate their deficit lead to different activation patterns (Fernandez et al., 2004).

### General discussion

Despite the methodological challenges of imaging overt language production (e.g., Gracco et al., 2005; Kemeny et al., 2005) this study has shown that the neural correlates of agrammatic continuous speech production can be reasonably investigated with an overt language production fMRI paradigm. This is in line with first studies on continuous speech production in healthy speakers (e.g., Kircher et al., 2005; Troiani et al., 2008; Grande et al., 2012) and in a single case pilot study with an aphasic speaker with lexical impairment (Tillmanns et al., 2011). Grande et al. (2012) and Tillmanns et al. (2011) aimed at explicitly imaging speech errors in contrast to errorless language production in picture description tasks. The present study was the first to focus on morpho-syntactic processing and agrammatic errors in a likewise paradigm. The statistical analysis of differences between the aphasic speakers' and the healthy participants' speech production in the 3W and NAT-condition revealed that the paradigm was suited for inducing agrammatic-like language production in healthy speakers even though the aphasic speakers in our study did not present with severe agrammatism. Although speech production elicited by picture description is not completely spontaneous (Prins and Bastiaanse, 2004), it is more naturalistic and involves more complex processes than single word or sentence production. Morpho-syntactic processes in speech production as they are required in natural communication cannot solely be studied in meta-linguistic tasks on word level. By using three approaches to investigate the activation pattern specific for morpho-syntactic processing in continuous language production we gained further insights into the neural basis of complete, complex and morphologically correct sentences and their agrammatic contrary. In healthy speakers (experiment 1), the structure-function mapping of unimpaired morpho-syntactic processes in language production and the investigation of the neural mechanisms underlying induced agrammatism (that is sentences with artificially reduced morpho-syntactic structures) revealed new insights into the activation patterns associated with selective aspects of morpho-syntax in spontaneous speech before brain injury, that are often impaired in agrammatism. The outcome provided the basis for the interpretation of the activation patterns associated with the same aspects of morpho-syntactic processing and the functional reorganization in five aphasic single cases with agrammatism. Combining fMRI with diffusion tensor imaging in future research might give interesting insights on the connectivity of the brain regions associated with morpho-syntactic processing in continuous language production, agrammatism, and functional reorganization (Saur et al., 2008; Friederici, 2009; Wilson et al., 2011; Griffiths and Marslen-Wilson, 2013). Thompson et al. (2010b) reported individual activation patterns in six agrammatic participants performing a sentence processing task pre and post treatment with down- and upregulations in several areas before and after the treatment. In our study, the results of the aphasic speakers also revealed individual activation patterns for agrammatic and unimpaired speech, probably due to patient variables discussed above. Variance due to possibly delayed hemodynamic response functions after stroke, which might have influenced the detection of activation in some areas (Bonakdarpour et al., 2007), cannot be excluded. Beyond that, Caplan et al. (2007) reported inherently different neural recruitment during syntax processing. By averaging aphasic participants' brain imaging data in a group study, the information about individual activation patterns would get lost (Rosen et al., 2000; Postman-Caucheteux et al., 2010). Then again group studies with aphasic speakers with different clinical profiles might reveal commonalities in brain modulation (e.g., Fridriksson et al., 2009) that might guide subsequent brain stimulation studies, e.g., with TMS (Devlin and Watkins, 2007; Naeser et al., 2011; Turkeltaub et al., 2011). Therefore, group studies on aphasic language processing should be carefully composed of aphasic participants, regarding their lesion size and location, time post onset, type and severity of their aphasic symptoms and their remaining language capacities (Thompson and den Ouden, 2008). For a full understanding of functional reorganization in the lesioned brain, common activation patterns of groups need to be augmented with details on activation patterns in individual cases.

### Conclusion

The present fMRI study has shown that the investigation of the neural correlates of agrammatic language production can be reasonably conducted with an overt language production task. The 3W-paradigm was suited for inducing agrammatic speech production in healthy speakers. Contrasting agrammatic with syntactically correct utterances elicited in a picture description task of both aphasic and healthy speakers revealed information on the neurobiological basis of morpho-syntactic processing in natural language production as well as in aphasia. In the group of healthy speakers, the pMTG and the inferior parietal cortex including the left AG and SMG were associated with greater morpho-syntactic demands in complete and complex CLUs. The intentional manipulation of morpho-syntactic structures and the omission of function words were associated with activation in the pMTG, the parietal lobule, the IFG, and MFG. During agrammatic and unimpaired language production the aphasic speakers revealed increased and decreased activation in the specific language areas involved in the healthy speakers' and areas associated with monitoring and attention. Effects in preserved and perilesional areas were always accompanied by activation in the non-lesioned hemisphere, reflecting compensational intra- and interhemispheric processes of dysfunctional areas directly or indirectly affected by the lesion. Some of these activation patterns seemed to successfully result in a syntactically correct sentence, while others seemed to remain unsuccessful resulting in an agrammatic utterance. Whether increased activation reflected compensational processes to overcome a struggle in language production or if it was the cause of it, could not be resolved in the scope of this paper. The same holds for the assumption that activation in some areas was not strong enough (decreased effects), causing the errors. These issues could be further investigated with repetitive transcranial magnetic stimulation (rTMS) or transcranial direct current stimulation (tDCS) applied to the identified areas (Devlin and Watkins, 2007; Winhuisen et al., 2007; Martin et al., 2009; Hamilton et al., 2010; Fridriksson, 2011; Meinzer et al., 2011; Naeser et al., 2011; Turkeltaub et al., 2012): Improved morpho-syntax after low frequency (inhibitory) rTMS or cathodal tDCS might suggest an increased activation in the cortical region of interest with a negative impact on language functions concerned. An enhancement in language capacities after high frequency TMS or anodal tDCS would indicate the accrual of agrammatic errors due to decreased activity. Individual patterns in the aphasic speakers' functional data indicate that lesion size and location, the remaining language capacities and the strategies of aphasic speakers to compensate their deficit lead to differences in the effects. These results suggest that group studies of activation patterns in aphasic recovery should be complemented by single-case analysis.

### Conflict of interest statement

The authors declare that the research was conducted in the absence of any commercial or financial relationships that could be construed as a potential conflict of interest.
