# Supplementary material for: The neural correlates of agrammatism: Evidence from aphasic and healthy speakers performing an overt picture description task
Source: Front Psychol. 2014 Mar 21;5:246. doi: 10.3389/fpsyg.2014.00246 (PMC3968764; doi:10.3389/fpsyg.2014.00246)
Supplement: Supplementary file 1 [file Presentation1.PDF]

1. Supplementary material

Table 1: Head movement during scanning

	Translation parameters									Rotation parameters								
	x-axis (mm)			y-axis (mm)			z-axis (mm)			pitch (°)			roll (°)			yaw (°)		
	range	mean	SD	range	mean	SD	range	mean	SD	range	mean	SD	range	mean	SD	range	mean	SD
A1	1.493	0.351	0.261	2.175	-0.839	0.308	2.473	-1.572	0.424	0.067	0.048	0.011	0.030	0.002	0.006	0.049	0.016	0.007
A2	1.254	-0.328	0.211	2.587	0.122	0.328	2.302	-0.850	0.438	0.043	0.002	0.008	0.043	0.019	0.007	0.042	-0.013	0.006
A3	0.675	0.039	0.089	1.764	0.226	0.271	3.891	-1.394	0.656	0.037	-0.002	0.004	0.028	0.004	0.004	0.022	-0.002	0.003
A4	1.608	-0.988	0.359	2.403	-0.156	0.292	3.982	-1.121	0.757	0.039	0.015	0.008	0.020	-0.008	0.005	0.019	-0.010	0.004
A5	1.317	-0.902	0.251	2.581	1.443	0.340	2.969	-2.188	0.355	0.037	0.025	0.006	0.021	-0.001	0.004	0.014	-0.003	0.003
Mean controls	1.963	-0.728	1.415	2.131	0.144	0.911	5.403	1.383	3.799	0.059	-0.003	0.034	0.047	-0.016	0.029	0.023	-0.009	0.012

A4: only 6 pictures included; SD: standard deviation

Table 2: Effect of syntactic completeness in the healthy speakers, reported at $P<.001_{uncorr}$, extend threshold k≥14 voxels based on Monte Carlo correction

Cluster size (voxels)	Local maximum in macroanatomical structure	MNI coordinates			T_{max}	Percent of overlap of cluster with cytoarchitectonical areas	
		x	y	z			
3W-simple-complete > 3W-simple-incomplete due to a missing verb							
143	L middle temporal gyrus	-62	-42	4	4.66		
133	L middle temporal gyrus	-58	-70	14	4.43	43.3	L IPC (PGp)[1]
						12.3	L IPC (PGa)[1]
115	L precuneus	-10	-60	48	4.64	25.8	L SPL (7A)[2]
						6.4	L SPL (7P)[2]
95	R anterior cingulate cortex	12	52	12	4.07		
32	R middle occipital gyrus	52	-78	6	3.61		cluster extends into the
						43.0	R IPC (PGp)[1]
32	L thalamus	-8	-4	4	4.05	48.0	L thalamus
27	R middle frontal gyrus	54	16	40	3.86	17.1	R area 44[3]

Cluster size (voxels)	Local maximum in macroanatomical structure	x	y	z	Tmax	Percent of overlap	Cytoarchitectonical area
25	L middle occipital gyrus	-34	-66	4	3.51		
22	L paracentral lobule	-6	-26	50	3.74	76.1	L area 4a[4]
						20.5	L area 6[5]
19	R middle temporal gyrus	62	-44	8	3.68		
15	L inferior parietal lobule	-56	-42	36	3.53	95.8	L IPC (PF)[1]
3W- simple-incomplete due to a missing verb > 3W-simple-complete							
not significant							

The coordinates (x,y,z) refer to anatomical MNI space. L: left; R: right; Tmax: maximum T value in the anatomical structure. Reference to cytoarchitectonic probability maps: [1]Caspers et al. (2006; 2008); [2]Scheperjans et al. (2008a; 2008b); [3]Amunts et al. (1999); [4]Geyer et al. (1996); [5]Geyer (2003); [6]Geyer et al. (1999; 2000); [7]Grefkes et al. (2001); [8]Rottschy et al. (2007); [9]Amunts et al. (2000); [10]Eickhoff et al. (2006a; 2006b); [11]Morosan et al. (2001; 2005); [12]Choi et al. (2006); [13]Amunts et al. (2005); [14]Kurth et al. (2010)

Table 3: Effect of complexity in the healthy speakers, reported at $P<.001_{uncorr}$, extend threshold k≥14 voxels based on Monte Carlo correction

Cluster size (voxels)	Local maximum in macroanatomical structure	MNI coordinates x	y	z	T_{max}	Percent of overlap of cluster with cytoarchitectonical areas	
NAT-complex-complete > NAT-simple-complete							
394	L middle cingulate cortex	0	-26	36	4.44		
303	L postcentral gyrus	-24	-36	58	5.79	33.4	L area 3b[6]
						18.2	L area 3a[6]
						17.8	L area 4p[4]
						10.0	L area 2[7]
167	L angular gyrus	-54	-70	32	4.35	60.9	L IPC (PGp)[1]
						18.6	L IPC (PGa)[1]
152	L inferior parietal lobule	-50	-54	46	4.15	88.4	L IPC (PFm)[1]
						6.3	L IPC (PGa)[1]
144	L cuneus	-10	-64	26	4.45		
57	thalamus	-6	4	-4	3.78	27.4	L thalamus
50	L middle cingulate cortex	-10	-4	34	4.29		
46	L lingual gyrus	-12	-84	-6	3.99	75.3	L hOC3v (V3v)[8]
						24.5	L area 18[9]

This is a provisional file, not the final typeset article

34	R supramarginal gyrus	52	-44	34	3.69	98.9	R IPC (PFm)[1]
24	R middle cingulate cortex	4	-2	44	3.87		cluster extends into the
						62.5	R area 6[5]
						14.1	L area 6[5]
21	L middle occipital gyrus	-38	-72	36	3.85		cluster extends into the
						65.5	L IPC (PGp)[1]
20	R inferior frontal gyrus	58	22	28	3.45	90.0	R area 45[3]
19	L precuneus	-8	-44	64	3.66	79.6	L area 4a[4]
						19.1	L SPL (5M)[2]
17	L superior medial gyrus	-10	34	52	3.92		
15	R superior frontal gyrus	18	-14	70	3.87	100.0	R area 6[5]
14	L middle occipital gyrus	-30	-86	34	3.82		cluster extends into the
						48.2	L IPC (PGp)[1]
NAT-simple-complete > NAT-complex-complete							
not significant							

For further details see legend of Table 2

Table 4: Effect of morphology in the healthy speakers, reported at $P<.001_{uncorr}$, extend threshold k≥14 voxels based on Monte Carlo correction

Cluster size (voxels)	Local maximum in macroanatomical structure	MNI coordinates			T_{max}	Percent of overlap of cluster with cytoarchitectonical areas	
		x	y	z			
NAT-simple-complete > 3W-simple-complete-morphological errors							
not significant							
3W-simple-complete-morphological errors > NAT-simple-complete							
653	L supramarginal gyrus	-56	-40	34	4.46	26.1	L IPC (PF)[1]
						24.7	L OP 4[10]
						9.7	L IPC (PFop)[1]
						8.8	L OP 1[10]
						6.3	L TE 1.2[11]
164	L middle temporal gyrus	-62	-44	6	5.47		
161	R middle frontal gyrus	36	-4	52	4.32	13.2	R area 6[5]

138	L inferior frontal gyrus	-38	34	4	4.62		
120	R postcentral gyrus	18	-30	60	4.97	36.5	R area 4p[4]
						33.8	R area 4a[4]
						18.9	R area 6[5]
115	R middle frontal gyrus	52	16	40	4.83	9.2	R area 44[3]
107	R anterior cingulate cortex	16	18	26	4.92		
105	L cerebellum	-8	-80	-18	3.87	57.4	L lobule VI (hem)
						10.5	L lobule VIIa crus I (hem)
						9.3	L lobule VI (vermis)
86	L inferior parietal lobule	-36	-50	46	4.19	38.8	L hIP1[12]
						33.3	L hIP3[12]
						10.8	L SPL (7A)[2]
						6.5	L area 2[7]
						6.3	L SPL (7PC)[2]
64	L middle frontal gyrus	-30	28	48	3.73		
60	L thalamus	-8	-4	4	4.34	53.9	L thalamus
55	L precentral gyrus	-54	6	30	3.95	36.6	L area 44[3]
						7.7	L area 6[5]
47	R frontal lobe, subgyral, white matter	34	32	14	3.90		
41	L inferior parietal lobule	-52	-50	52	3.73	57.3	L IPC (PFm)[1]
						29.0	L IPC (PF)[1]
41	L lingual gyrus	-14	-60	-4	3.69	46.6	L area 18[9]
						23.2	L hOC3v (V3v)[8]
						16.2	L lobule VI (hem)
						10.1	L hOC4v (V4)[8]
40	R SMA	12	-16	58	3.95	93.4	R area 6[5]
40	L postcentral gyrus	-22	-42	66	3.98	45.9	L SPL (5L)[2]
						26.6	L area 3b[6]
						14.7	L area 2[7]
						11.3	L area 1[6]
38	R cerebellum	16	-50	-26	3.78	67.4	R lobule VI (hem)
						28.0	R lobule V
35	L middle temporal gyrus	-46	-40	-2	3.95		

This is a provisional file, not the final typeset article

34	L inferior parietal lobule	-32	-74	48	4.08	62.5	L IPC (PGa)[1]
						26.5	L IPC (PGp)[1]
						5.5	L SPL (7A)[2]
34	L precentral gyrus	-32	-10	44	4.00	8.5	L area 6[5]
28	L middle temporal gyrus	-62	-4	-10	3.78	17.0	L TE 3[11]
26	L middle frontal gyrus	-32	16	26	3.45		
25	L SMA	-6	14	52	3.74	65.0	L area 6[5]
21	R Rolandic operculum	44	4	12	3.75		
18	L postcentral gyrus	-34	-44	66	3.68	71.5	L area 1[6]
						19.4	L area 2[7]
18	R parahippocampal gyrus	34	-20	-24	3.95	76.4	R hipp (CA)[13]
						20.8	R hipp (SUB)[13]
17	L middle occipital gyrus	-38	-74	38	3.87		cluster extends into the
						98.5	L IPC (PGp)[1]
14	L postcentral gyrus	-56	-8	44	3.56	62.5	L area 6[5]
						35.7	L area 1[6]
14	R inferior frontal gyrus	46	18	26	3.36		

For further details see legend of Table 2

Table 5: Effect of morpho-syntactic planning in the healthy speakers, reported at $P<.001_{uncorr}$, extend threshold k≥14 voxels based on Monte Carlo correction

Cluster size (voxels)	Local maximum in macroanatomical structure	MNI coordinates			T_{max}	Percent of overlap of cluster with cytoarchitectonical areas	
		x	y	Z			
NAT-pauses/interjections between CLUs >3W- pauses/interjections between CLUs							
92	R angular gyrus	48	-56	26	3.45	43.8	R IPC (PFm)[1]
						25.5	R IPC (PGa)[1]
						7.6	R IPC (PFcm)[1]
64	L middle temporal gyrus	-52	-56	22	4.13	41.4	L IPC (PGa)[1]
						4.9	L IPC (PGp)[1]
38	R angular gyrus	52	-68	32	3.85	88.2	R IPC (PGp)[1]
						10.5	R IPC (PGa)[1]

35	R precuneus	36	-58	30	3.76		
30	R temporal lobe, subgyral, white matter	46	-2	-20	3.83	17.5	R insula (Id1)[14]
24	R precuneus	16	-40	36	4.01		
3W- pauses/interjections between CLUs > NAT-pauses/interjections between CLUs							
937	L inferior frontal gyrus	-44	22	16	5.16	21.3	L area 44[3]
80	L temporal lobe, subgyral, white matter	-46	-38	-2	3.95		
52	L superior frontal gyrus	-18	52	24	4.30		
49	L frontal lobe, subgyral, white matter	-34	-16	30	3.69		
46	L sublobar, extra-nuclear, white matter	-18	-8	16	3.98	32.9	L thalamus
31	L precentral gyrus	-46	-6	54	4.09	99.6	L area 6[5]
30	R inferior frontal gyrus	42	12	14	3.70	21.7	R area 44[3]
24	R cerebellum	14	-80	-26	3.60	51.0	R lobule VIIa crus I (hem)
						49.0	R lobule VI (hem)
21	L inferior occipital gyrus	-46	-72	-8	3.55	5.4	L hOC5[8]
14	L superior frontal gyrus	-24	-4	68	3.79	92.0	L area 6[5]

For further details see legend of Table 2

This is a provisional file, not the final typeset article

References

Amunts, K., Kedo, O., Kindler, M., Pieperhoff, P., Mohlberg, H., Shah, N. J., Habel, U., Schneider, F., and Zilles, K. (2005). Cytoarchitectonic mapping of the human amygdala, hippocampal region and entorhinal cortex: intersubject variability and probability maps. Anatomy and Embryology 210, 343–352. doi: 10.1007/s00429-005-0025-5.

Amunts, K., Malikovic, A., Mohlberg, H., Schormann, T., and Zilles, K. (2000). Brodmann's areas 17 and 18 brought into stereotaxic space - where and how variable? NeuroImage 11, 66–84. doi:10.1006/nimg.1999.0516.

Amunts, K., Schleicher, A., Bürgel, U., Mohlberg, H., Uylings, H. B. M., and Zilles, K. (1999). Broca' s region revisited: Cytoarchitecture and intersubject variability. The Journal of Comparative Neurology 412, 319–341. doi:10.1002/(SICI)1096-9861(19990920)412:2<319::AID-CNE10>3.0.CO;2-7.

Caspers, S., Eickhoff, S. B., Geyer, S., Scheperjans, F., Mohlberg, H., Zilles, K., and Amunts, K. (2008). The human inferior parietal lobule in stereotaxic space. Brain Structure & Function 212, 481–495. doi:10.1007/s00429-008-0195-z.

Caspers, S., Geyer, S., Schleicher, A., Mohlberg, H., Amunts, K., and Zilles, K. (2006). The human inferior parietal cortex: Cytoarchitectonic parcellation and interindividual variability. NeuroImage 33, 430–448. doi:10.1016/j.neuroimage.2006.06.054.

Choi, H.-J., Zilles, K., Mohlberg, H., Schleicher, A., Fink, G. R., Armstrong, E., and Amunts, K. (2006). Cytoarchitectonic identification and probabilistic mapping of two distinct areas within the anterior ventral bank of the human intraparietal sulcus. Journal of Comparative Neurology 495, 53–69. doi:10.1002/cne.20849.

Eickhoff, S. B., Amunts, K., Mohlberg, H., and Zilles, K. (2006). The human parietal operculum. II. Stereotaxic maps and correlation with functional imaging results. Cerebral Cortex 16, 268–279. doi:10.1093/cercor/bhi106.

Eickhoff, S. B., Schleicher, A., Zilles, K., and Amunts, K. (2006). The human parietal operculum. I. Cytoarchitectonic mapping of subdivisions. Cerebral Cortex 16, 254–267. doi:10.1093/cercor/bhi105.

Geyer, S. (2003). The Microstructural Border Between the Motor and the Cognitive Domain in the Human Cerebral Cortex. Wien: Springer.

Geyer, S., Ledberg, A., Schleicher, A., Kinomura, S., Schormann, T., Bürgel, U., Klingberg, T., Larsson, J., Zilles, K., and Roland, P. E. (1996). Two different areas within the primary motor cortex of man. Nature 382, 805–807. doi:10.1038/382805a0.

Geyer, S., Schleicher, A., and Zilles, K. (1999). Areas 3a , 3b, and 1 of human primary somatosensory cortex. NeuroImage 10, 63–83. doi:10.1006/nimg.1999.0440.

Geyer, S., Schormann, T., Mohlberg, H., and Zilles, K. (2000). Areas 3a, 3b, and 1 of human primary somatosensory cortex. Part 2. Spatial normalization to standard anatomical space. NeuroImage 11, 684–696. doi:10.1006/nimg.2000.0548.

Grefkes, C., Geyer, S., Schormann, T, Roland, P., and Zilles, K. (2001). Human somatosensory area 2: Observer-independent cytoarchitectonic mapping, interindividual variability, and population map. NeuroImage 14, 617–31. doi:10.1006/nimg.2001.0858.

Kurth, F., Eickhoff, S. B., Schleicher, A., Hoemke, L., Zilles, K., and Amunts, K. (2010). Cytoarchitecture and probabilistic maps of the human posterior insular cortex. Cerebral Cortex 20, 1448–1461. doi:10.1093/cercor/bhp208.

Morosan, P., Rademacher, J., Schleicher, A., Amunts, K., Schormann, T., and Zilles, K. (2001). Human primary auditory cortex: Cytoarchitectonic subdivisions and mapping into a spatial reference system. NeuroImage 13, 684–701. doi:10.1006/nimg.2000.0715.

Morosan, P., Schleicher, A., Amunts, K., and Zilles, K. (2005). Multimodal architectonic mapping of human superior temporal gyrus. Anatomy and Embryology 210, 401–406. doi:10.1007/s00429-005-0029-1.

Rottschy, C., Eickhoff, S. B., Schleicher, A., Mohlberg, H., Kujovic, M., Zilles, K., and Amunts, K. (2007). Ventral visual cortex in humans: Cytoarchitectonic mapping of two extrastriate areas.

Scheperjans, F., Eickhoff, S. B., Hömke, L., Mohlberg, H., Hermann, K., Amunts, K., and Zilles, K. (2008). Probabilistic maps, morphometry, and variability of cytoarchitectonic areas in the human superior parietal cortex. Cerebral Cortex 18, 2141–2157. doi:10.1093/cercor/bhm241.

Scheperjans, F., Hermann, K., Eickhoff, S. B., Amunts, K., Schleicher, A., and Zilles, K. (2008). Observer-independent cytoarchitectonic mapping of the human superior parietal cortex. Cerebral Cortex 18, 846–867. doi:10.1093/cercor/bhm116.
